# Social determinants of health and rehabilitation service areas: an urban and rural mediation analysis

**DOI:** 10.3389/fpubh.2025.1562610

**Published:** 2025-06-18

**Authors:** Sanghun Nam, Susanne Schmidt, Julianna M. Dean, Alex F. Bokov, Timothy A. Reistetter

**Affiliations:** ^1^Department of Occupational Therapy, The University of Texas Health Science Center at San Antonio, San Antonio, TX, United States; ^2^Department of Population Health Sciences, The University of Texas Health Science Center at San Antonio, San Antonio, TX, United States; ^3^Department of Health Studies, Sul Ross State University, Alpine, TX, United States; ^4^University of Texas Medical Branch at Galveston, Galveston, TX, United States

**Keywords:** social determinants of health, rehabilitation, healthcare disparities, urban health, rural health

## Abstract

**Introduction:**

Social determinants of health significantly shape community discharge (CD) rates in post-acute rehabilitation settings. Additionally, healthcare disparities between urban and rural regions in the United States can affect these discharge rates. These disparities underscore the critical need to understand how social, economic, educational, and healthcare-related factors influence community discharge outcomes to guide equitable healthcare strategies.

**Methods:**

This observational, cross-sectional study analyzed 40,476 ZIP code tabulation area (ZCTA)-level data points linked to rehabilitation service areas and Agency for Healthcare Research and Quality datasets. Exploratory and confirmatory factor analyses identified five social determinants of health domains—social, economic, educational, physical infrastructure, and healthcare—which were assessed using structural equation modeling to evaluate their direct and mediated effects on community discharge rates.

**Results:**

Significant disparities in community discharge rates were observed across urban and rural areas. Urban areas exhibited lower community discharge rates, influenced by higher social and economic deprivation and limited English proficiency. Conversely, rural areas demonstrated higher rates, attributed to areal social, economic, and education characteristics. Key factors affecting community discharge outcomes included economic inequities, limited healthcare access, and transportation barriers.

**Conclusion:**

Targeted interventions addressing economic inequities, healthcare access, and transportation challenges are essential to improving community discharge outcomes. These findings inform policy and healthcare practices aimed at fostering equitable rehabilitation services and optimizing community reintegration.

## Introduction

Social determinants of health (SDH) are main drivers of inequities in healthcare. Defined as the conditions and contexts where people live and work, these determinants represent a complex interaction of social structures, economic systems, and physical environments. Federal and international policymakers and agencies have reported on the importance of SDH as powerful contributors to healthcare utilization and quality of care outcomes (1, 2). SDH are reported to account for 30%−55% of health outcomes (3) and are associated with higher healthcare expenditures (4).

Even though SDH represent a large portion of health outcomes and account for considerable costs, there is no agreed upon theoretical model for SDH. The Dahlgren and Whitehead model of population health visually shows the inter-related layers of social determinant domains on health which has been utilized internationally (5). A common classification scheme by policy makers characterizes factors into categories or domains of social determinants. For example, the Agency for Healthcare Research and Quality (AHRQ) breaks SDH into five domains: economic, education, physical environment, social context, and healthcare (6). Healthy People 2030 domains are similar: economic stability, education access and quality, neighborhood and built environment, social and community context, and healthcare access and quality (7). Additionally, multiple indices exist that combine different SDH measures. Some of these are the Area Deprivation Index (8), the Distressed Community Index (9), the Social Deprivation Index (10), and the Social Vulnerability Index (11). These indices are calculated in various ways with different purposes in mind resulting in the healthcare context domain being largely absent. A review of existing indices is beyond the scope of this paper. These challenges have resulted in limitations in SDH research as it relates to the healthcare domain and healthcare outcomes.

There is growing recognition that both individual and community level social determinates impact health outcomes across healthcare sectors and systems (12–16). One healthcare sphere where community context plays a role in outcomes is post-acute rehabilitation. Post-acute rehabilitation is an important component of the healthcare system providing services for a myriad of health-related issues. In 2022, post-acute care accounted for $57.8 billion in health care spending across rehabilitation service venues of long-term care hospitals, inpatient rehabilitation facilities, skilled nursing facilities, and home health agencies which each operate under different admission and payment structures (17). Moreover, substantial geographic variation in the distribution of these service venues exists which influences rehabilitation outcomes (18–21).

The National Institutes of Health report, Moving Rehabilitation Research Forward discusses the complex interaction between the environment and health outcomes suggesting the need for refined approaches and policies to improve care (22). Like other sectors of healthcare which have noted disparities linked to social determinants (23), rehabilitation research has found disparities in functional status and hospital readmission connected to the SDH domains: socioeconomic, education, and neighborhood quality (24–29).

Rehabilitation service areas (RSA) were constructed to aid in the examination and understanding of geographic differences in care and subsequent rehabilitation outcomes. Like hospital and primary care service areas, RSAs are post-acute care utilization areas for rehabilitation service use in the US. There are 1,711 RSAs nationally built from ZIP code tabulation areas (ZCTA) based on patterns of rehabilitation care (30, 31). RSAs are a tool to describe and analyze resource availability utilization, care patterns, and gaps in rehabilitation care.

A prominent rehabilitation goal is to help return someone to their home with the ability to function independently in the community. This goal has driven the development of successful community discharge (CD) as a quality measure for post-acute care rehabilitation. The Centers for Medicare and Medicaid Services defined community discharge as remaining in the community for 31 days without an unplanned hospital readmission. The recognition of successful community discharge as a quality measure for post-acute rehabilitation is inherently linked to environmental factors (32, 33) and likely area level social determinants of health. Moreover rural and urban differences in rehabilitation use suggest potential disparities (34) and the influence of SDH. In a study of rehabilitation use between urban and rural areas, researchers found significant differences in the use of skilled nursing and home health services (35). The impact of area level social determinant on rural and urban rehabilitation use remains understudied. Although there is growing rehabilitation research on SDH, many of these studies evaluate select variables, are often limited by diagnosis, and do not evaluate the panoply of area characteristics and resources.

This study aims to bridge critical gaps in understanding the influence of area-level SDH on community discharge outcomes within post-acute care rehabilitation settings. By leveraging comprehensive datasets at the ZCTA level and integrating RSA-specific service patterns, this research identifies how community-level factors intersect with healthcare access and utilization. Such analyses are pivotal for developing tailored interventions to enhance post-acute care outcomes and reduce disparities in rehabilitation efficacy. Ultimately, this work seeks to inform policymakers and healthcare stakeholders about strategies to optimize community reintegration, advancing equity and quality in healthcare delivery systems.

## Methods

### Study design, setting, and data sources

This was an observational, cross-sectional secondary data analysis of US data from two main sources. First, we used the publicly available Social Determinants of Health Database from the Agency for Healthcare Research and Quality (AHRQ) (6). This database was created to facilitate research and ultimately improve health outcomes. It provides a single file with a range of variables across several domains that can easily be linked to other data (6). We used the 2016 ZIP Code Data, which includes 5-year estimates from the 2012–2016 American Community Survey (ACS) at the ZCTA level.

Second, we used a crosswalk dataset that contained three variables: (1) rehabilitation service areas (RSAs) (30), (2) ZCTA, and (3) community discharge rates per RSA. This dataset contains 1,711 observations, corresponding to the 1,711 RSAs across the United States and is publicly available for use by all researchers (36). Whereas, RSAs are geographic boundaries similar in concept to Primary Care Service Areas and Hospital Service Areas, RSAs represent the unique patterns of post-acute care services that other small-area boundaries do not capture. We merged the crosswalk dataset and the AHRQ dataset through ZCTA variables and extracted SDH and community discharge rate from the AHRQ dataset. The RSA and community discharge data were developed using 2013–2015 national Medicare claims data, which temporally aligns with the 2012–2016 American Community Survey period used in the AHRQ SDH dataset.

### Variables

#### Social determinants of health

The AHRQ SDH Database 2016 ZCTA Data contains 313 total variables that includes the following contexts form the ACS: social, economic, education, physical infrastructure, and healthcare. The 2016 AHRQ Codebook indicates the following number of variables per domain: social (123 variables), economic (80 variables), education (10 variables), physical infrastructure (69 variables), and healthcare (31 variables) (6).

From these variables, an expert panel experienced with older adult health outcomes selected variables thought to affect successful community discharge. This expert panel included professionals from demography, population health, data science, rehabilitation science, and a rehabilitation clinician. The panel arrived at 34 variables present in the 2016 data at the ZCTA level. The 34 ACS variables finally selected represent the percentage at the ZCTA levels (Supplementary Appendix S1). By domain, the following variables remained: social (10 variables), economic (six variables), education (three variables), physical infrastructure (nine variables), and healthcare (six variables).

A multidisciplinary expert panel consisting of professionals in demography, rehabilitation science, data science, and public health convened weekly over a 6-week period to review and select variables from the AHRQ SDH dataset. Variables were chosen based on two primary criteria: (1) percentage-based metrics to ensure comparability across ZCTAs, and (2) conceptual relevance to one of the five major SDH domains—social, economic, education, physical infrastructure, and healthcare. Although a formal Delphi process was not used to identify variables the expert panel engaged in structured discussions of SDH domains to ensure content validity and representation.

#### Community discharge

The community discharge (CD) rate is a metric designed to assess the rate at which patients are discharged to and remain in the community for 31 days without an unplanned readmission to the hospital. This metric is consistent with CMS measurement specifications for post-acute care rehabilitation (37, 38). In this study, the CD rate variable in the crosswalk dataset was merged into the AHRQ dataset by the Zip Code Tabulation Area (ZCTA) variable.

#### Rural-Urban commuting area (RUCA)

The Rural-Urban commuting area (RUCA) system classifies areas based on commuting patterns and population density. The RUCA system divides areas into four categories based on proposed guidelines (39). Urban core areas are highly urbanized, characterized by high population density and excellent access to resources and services. Suburban areas, located on the outskirts of cities, have dense populations and strong commuting connections to urban cores. Large rural areas are rural regions with relatively higher populations and moderate commuting ties to urban centers, whereas small town/rural areas include small communities and remote rural regions with limited commuting flows and restricted access to resources.

These classifications are well-suited for explaining variations in health outcomes and service accessibility across geographic areas (40). In this study, RUCA was particularly applied to analyze the mediating effect of service accessibility on the relationship between the five domains of SDH and successful community discharge. By leveraging RUCA, the study identified how regional characteristics indirectly influence rehabilitation outcomes and successful community reintegration, mediated by SDH domains such as economic, social, educational, physical, and healthcare contexts.

### Study size

We utilized a merged dataset derived from the crosswalk dataset and the AHRQ dataset, using the ZCTA variable as the key for merging. The original AHRQ dataset consisted of 40,824 observations. Through the ZCTA variable, the RSA, and CD rate variables from the crosswalk dataset were incorporated into the AHRQ dataset. During this process, 348 ZCTAs were excluded due to missing a RUCA classification or values for one or more of the 34 selected SDH variables. In total, 348 responses (0.85% of the original dataset) were excluded, resulting in 40,476 ZCTAs included in the final analysis. Moreover, we conducted a sensitivity analysis using inclusion status as the outcome and SDH variables as predictors. None of the variables were significantly associated with inclusion, suggesting that the exclusion process did not introduce meaningful bias across the SDH characteristics examined.

### Statistical analysis

#### Exploratory factor analysis (EFA)

In this study, we conducted an exploratory factor analysis (EFA) using 34 ACS variables initially selected to explore the latent factor structure among variables. All analyses were conducted using Mplus software version 8.4, applying Maximum Likelihood estimation with Robust standard errors (MLR) to address data non-normality (41). The MLR estimator also accommodates missing data through full information maximum likelihood under the assumption of data missing at random, allowing all available information to be utilized in the estimation process. The scree plot test was used to determine the number of variables by identifying the elbow point on the graph for the factors with eigenvalues >1. These methods are widely used to determine the number of factors, and to select the factors that explain the most variance in the data. In the analysis process, Mplus's default Geomin rotation (oblique rotation) was applied to allow for correlations between factors. The Geomin rotation was chosen over alternatives like Promax to align with expected interdependence among the latent variables in the dataset. Additionally, only items with factor loadings of 0.3 or higher were included in the subsequent confirmatory factor analysis (CFA), based on previous research suggesting that this threshold is sufficient. While some recommend a higher threshold such as 0.40 for large samples, the 0.30 threshold was deemed appropriate to capture conceptually relevant items given the multidimensional nature of the SDH constructs (42, 43).

#### Confirmatory factor analysis (CFA)

In this study, CFA was conducted to validate the factor structure derived from the EFA. The variance of the latent variables was fixed at one to ensure model identification, adding constraints for proper model specification. Correlations between factors were freely estimated to interpret factor loadings by fixing the relative size of each factor and to evaluate the relationships between factors. Similar to the EFA, only items with factor loadings of 0.3 or higher were included in the final analysis (43). Modification indices were reviewed to identify potential areas of misfit, but modifications were only made when they were theoretically justified to maintain the integrity of the model. In addition, standardized residuals were inspected to confirm that no large discrepancies existed between observed and model-implied covariances.

#### Structural equation modeling (SEM) regression

In this study, structural equation modeling (SEM) regression was conducted using the final variables selected through CFA to investigate the relationships between variables. The SEM analysis employed a multiple-factor model to evaluate the relationships among latent variables. The key latent variables included in the analysis were social factors, economic factors, education factors, physical infrastructure factors, and healthcare factors. We analyzed their effects on the dependent variable, CD rate (44). Bootstrapping methods were applied to estimate the confidence intervals for indirect effects, providing more robust inferences regarding mediation effects. Additionally, modification indices were reviewed to address potential areas of misfit, and model refinements were made iteratively where theoretically justified.

#### SEM mediation

In this study, mediation analysis using SEM was conducted to evaluate the indirect effects between the independent and dependent variables, employing the final variables selected through CFA. The SEM mediation analysis assessed both the direct and indirect effects of social factors, economic factors, education factors, physical infrastructure factors, and healthcare factors on the dependent variable, CD rate, through the mediator variable, RUCA. Specifically, RUCA served as a mediator in the relationship between the independent variables and the dependent variable, analyzing how the independent variables indirectly influenced CD rates. In the mediation model, RUCA classification was treated as an observed categorical mediator representing four distinct geographic contexts (urban core, suburban, large rural, and small town/rural).

The use of RUCA classification as a mediator is grounded in theories of urban health ecology and spatial health inequality, which posits that geographic context—particularly the degree of urbanization—influences how social and structural determinants of health translating into healthcare access and outcomes (45). Urban–rural distinctions shape service availability, care-seeking behaviors, and environmental constraints, positioning the RUCA classification as a conceptually grounded mediator through which area-level social determinants of health influence community discharge outcomes —consistent with theories of urban health ecology and spatial health inequality (46).

The significance of the mediation effects was tested using the bootstrapping method, which involved repeatedly resampling the data to estimate the confidence intervals of the indirect effects. This method enhances reliability by accounting for the uncertainty in the sample distribution (41). The significance of the indirect paths was confirmed through bootstrapping, and the mediation effects were evaluated based on the significant *p*-values derived from the estimated standard errors (47).

We calculated the indirect effects as the product of the a-path (SDH to RUCA) and the b-path (RUCA to CD). Additionally, to determine the total effect, we summed the direct and indirect effects. Finally, to assess the mediation effect, we divided the indirect effect by the total effect and expressed the extent of mediation as a percentage.

#### Model fit

In this study, the model fit of each model was assessed using the following indices: root mean square error of approximation (RMSEA), comparative fit index (CFI), Tucker–Lewis index (TLI), and standardized root mean square residual (SRMR). The criteria for each fit index are as follows. First, RMSEA is an index for evaluating model fit, where a value of 0.06 or lower indicates good fit, and values below 0.08 are considered acceptable (48). Second, CFI and TLI values above 0.95 indicate good fit, while values above 0.90 are deemed acceptable (48, 49). Third, SRMR values below 0.08 represent good fit, and this criterion was used to evaluate the SRMR values in this study (48). It is important to note that these fit indices may vary slightly depending on the complexity of the model and the sample size, which was considered in the interpretation of the results.

## Results

### Exploratory factor analysis (EFA)

As the first step of this study, an EFA was conducted using 34 ACS variables categorized in the initial classification. The results are as follows (Supplementary Appendix S2). The Social factor was divided into two components: the first factor included S5: does not speak English well or at all (λ = 0.860), S9 (λ = 0.913), and S10 (λ = 0.989), while the second factor included S4: single parent families (λ = 0.490), S6 (λ = 0.893), and S7 (λ = 0.969; *RMSEA* = 0.068, *CFI*/*TLI* = 0.909/0.843, and *SRMR* = 0.053). The economic factor was initially divided into three components, but after excluding duplicated items, it was ultimately classified into two factors. The first factor included EC1: under 3.99 of the poverty threshold (λ = 0.556) and EC3: not in labor force (λ = 0.701), while the second factor included EC2: household income < $24,999 (λ = 0.513), EC4: Asian population below poverty level (λ = 0.384), EC5: Black or African American population below poverty level (λ = 0.432), and EC6: some other race population below poverty level (λ = 0.433; *RMSEA* = 0.000, *CFI*/*TLI* = 1.000/1.000, and *SRMR* = 0.000). The education factor was grouped into a single factor, including ED1: a bachelor's degree (λ = 0.825), ED2: master's or doctorate (λ = 0.784), and ED3: only high school diploma (λ = −0.840; *RMSEA* = 0.000, *CFI*/*TLI* = 1.000/1.000, and *SRMR* = 0.000). The physical infrastructure factor was divided into two components: the first factor included PI2: housing units lacking compete kitchen facilities (λ = 0.867) and PI3: housing units lacking plumbing facilities (λ = 0.947), and the second factor included PI5: housing units with no vehicle available (λ = 0.916), PI6: workers taking public transportation, excluding taxicab (λ = 0.664), PI7: works taking taxicab, motorcycle or other means to work (λ = 0.365), PI8: workers walking to work (λ = 0.492), and PI9: workers in households with no vehicle available (λ = 0.960; *RMSEA* = 0.039, *CFI*/*TLI* = 0.950/0.905, and *SRMR* = 0.026). Finally, the Healthcare factor was also divided into two components: the first factor included H3: Medicare, Medicaid, TRICARE/military, U.S Department of Veterans Affairs coverage (λ = 0.902) and H6: no health insurance coverage (λ = 0.339), while the second factor included H1: other private-only health insurance combinations (λ = 0.611) and H5: TRICARE/military or VA health insurance coverage only (λ = 0.969; *RMSE* = 0.035, *CFI*/*TLI* = 0.937/0.763, and *SRMR* = 0.017).

### Confirmatory factor analysis (CFA)

In this study, CFA was conducted to confirm the validity of the factor structure identified in the EFA, allowing for correlations between the nine factors. The results of the CFA analysis are as follows (Supplementary Appendix S3) For the social factor, the first factor showed factor loadings ranging from 0.859 to 0.985, indicating strong associations, while the second factor had moderate to strong loadings ranging from 0.459 to 0.977. For the economic factor, the first factor displayed loadings between 0.445 and 0.834, and the second factor showed relatively lower loadings between 0.380 and 0.442, suggesting a weaker association. The education factor was represented by a single factor, with loadings of 0.801 and a negative factor loading of −0.794, which may indicate reverse coding or a differing pattern in the data. For the physical infrastructure factor, the first factor exhibited strong loadings between 0.884 and 0.931, and the second factor showed loadings ranging from 0.378 to 0.945. For the healthcare factor, the first factor had loadings between 0.572 and 0.989, indicating variability in factor strength, while the second factor displayed lower loadings from 0.428 to 0.599. Nonetheless, all factor loadings exceeded the threshold of 0.300, and thus all factors were retained for further analysis. The model fit indices for the CFA were as follows: *RMSE* = 0.058, *CFI*/*TLI* = 0.803/0.754, and *SRMR* = 0.075.

Table 1 presents descriptive statistics for the final items selected through CFA, showing their distribution across different residential regions. The most notable variables by region are as follows: in urban core areas, the most notable characteristic was the higher percentage of foreign-born individuals (S9: 10.66%) compared to other regions. Additionally, the use of public transportation was highest here (PI6: 4.21%). In Suburban areas, the highest proportion of the population under 3.99 of the poverty thresholds was observed (EC1: 72.30%). Furthermore, a significant percentage of the population had only a high school diploma (ED4: 36.92%). In large rural areas, the population without labor force participation was notably high (EC3: 44.01%). This region also had the highest percentage of households lacking kitchen facilities (PI2: 5.82%). In small town/rural areas, the highest percentage of workers walked to work (PI8: 5.85%), and the region also had the greatest proportion of households lacking plumbing facilities (PI3: 7.59%).

**Table 1:** Social determinants of health demographic characteristics by region variables (*N* = 40,476).

**Variables**	**Urban core (*****n*** = **24,179)**		**Suburban (*****n*** = **5,578)**		**Large rural (*****n*** = **3,922)**		**Small town/rural (*****n*** = **6,797)**	
	**M**	**SD**	**M**	**SD**	**M**	**SD**	**M**	**SD**
Community discharge	54.45	3.26	55.62	3.14	55.92	3.11	55.64	3.54
% of families with children that are single-parent families (S4)	30.27	19.53	29.10	19.00	28.74	19.35	25.35	21.56
% of population that is minority (S6)	18.99	20.90	9.53	16.23	8.93	15.47	4.96	12.26
% of householders who minority (S7)	18.01	20.91	9.65	16.64	9.78	16.91	8.27	19.15
% of population that does not speak English at all or well (S5)	3.12	5.39	1.38	3.55	1.33	3.73	1.04	3.03
% of population that is foreign-born (S9)	10.66	11.48	3.95	5.85	3.63	6.03	3.35	5.66
% of population who are not U.S. citizens (S10)	5.23	6.66	2.11	4.02	1.99	4.46	1.67	3.97
% of population under 3.99 of the poverty thresholds (EC1)	62.63	19.19	72.30	13.94	74.06	13.02	72.15	14.25
% of population not in labor force (EC3)	37.56	10.81	42.20	11.65	44.01	12.37	42.70	13.41
% of Asian population below poverty level (EC4)	11.58	19.30	8.20	20.54	8.21	22.43	5.91	21.02
% of Black or African American population below poverty level (EC5)	21.28	22.49	20.88	28.09	20.86	30.30	13.54	29.02
% of some other race population below poverty level (EC6)	17.76	24.60	14.47	26.43	13.69	28.19	9.14	25.11
% of population with a bachelor's degree (ED2)	18.10	10.22	11.83	7.68	11.19	7.26	12.62	8.74
% of population with master's or doctorate (ED3)	11.60	10.19	6.32	5.67	5.79	5.42	6.06	6.55
% of population with only high school diploma (ED4)	29.10	12.36	36.92	11.38	37.74	11.04	37.13	11.79
% of population housing units lacking compete kitchen facilities (PI2)	3.17	4.56	4.75	5.35	5.82	6.38	7.32	9.09
% of population housing units lacking plumbing facilities (PI3)	2.45	4.35	4.37	5.63	5.63	7.09	7.59	10.39
% of housing units with no vehicle available (PI5)	8.85	11.74	5.61	5.33	6.11	5.75	6.08	10.96
% of workers taking public transportation, excluding taxicab (PI6)	4.21	9.05	0.54	1.72	0.50	1.66	0.63	3.16
% of works taking taxicab, motorcycle or other means to work (P7)	1.92	2.79	1.46	2.79	1.69	3.55	2.35	6.57
% of workers walking to work (PI8)	4.10	8.69	2.87	5.47	3.74	6.84	5.85	10.32
% of workers in households with no vehicle available (PI9)	4.38	9.14	2.11	3.22	2.35	4.59	3.16	10.07
% of population other private-only health insurance combinations (H3)	0.43	1.32	0.33	0.90	0.32	1.55	0.29	1.39
% of population with Medicare, Medicaid, TRICARE/military, U.S Department of Veterans Affairs coverage (H5)	1.52	4.88	1.28	3.78	1.22	4.40	1.25	5.13
% of population with TRICARE/military or VA health insurance coverage only (H7)	22.18	12.28	25.09	11.62	26.53	12.22	24.76	13.68
% of population with no health insurance coverage (H8)	10.91	7.51	11.84	7.56	12.14	8.33	12.40	9.70

### SEM regression

Table 2 analyzes the relationship between the nine factors and CD rate based on the SEM regression results. First, with the exception of the first factor of physical infrastructure (*p* = 0.204) and the first factor of healthcare (*p* = 0.111), all factors showed significant differences in their effects on CD rate. For the social factor, both factors were negatively associated with CD rate, with estimates of −0.052 and −0.029, respectively. This refers to that as the proportions of social variables such as single-parent families, foreign-born individuals, and minority populations increase, the CD rate decreases. The economic factor had contrasting results. The first factor exhibited a strong negative relationship with CD rate (β = −0.261), indicating that increases in variables like the percentage of the population below the poverty threshold and the percentage of the population not in the labor force are associated with a decrease in CD rate. Conversely, the second factor had a positive association (β = 0.093), indicating that as other economic indicators, such as the percentage of the population below the poverty level for specific racial groups, increase, CD rate may increase. The educational factor showed a substantial negative effect on CD rate (β = −0.444). This result points to that as the proportion of the population with higher education levels increases, CD rate tends to decrease. For the physical infrastructure factor, the second component showed a small negative relationship with CD rate (β = −0.031), indicating that increases in variables like the percentage of workers walking to work are associated with a slight reduction in CD rate. Lastly, the second component of the healthcare factor had a positive relationship with CD rate (β = 0.044), suggesting that as the percentage of the population covered by specific health insurance types, such as TRICARE or VA coverage, increases. The model fit indices for the SEM regression analysis were as follows: *RMSE* = 0.057, *CFI*/*TLI* = 0.806/0.754, and *SRMR* = 0.073.

**Table 2:** SEM regression model results for effects of social, economic, educational, physical infrastructure, and healthcare factors on community discharge rate.

**Factor**	**Community discharge rate**		
	**Estimate (standard)**	**SE**	***p*** **-value**
Social_1	−0.052	0.011	< 0.0001
Social_2	−0.029	0.009	0.001
Economic_1	−0.261	0.042	< 0.0001
Economic_2	0.093	0.027	0.001
Educational	−0.444	0.053	< 0.0001
Physical infrastructure_1	0.018	0.014	0.204
Physical infrastructure_2	−0.031	0.009	0.001
Healthcare_1	0.020	0.012	0.111
Healthcare_2	0.044	0.020	0.024
Model fit	RMSEA	CFI/TLI	SRMR
	0.057	0.806/0.754	0.073

### SEM mediation

**Figure 1:**
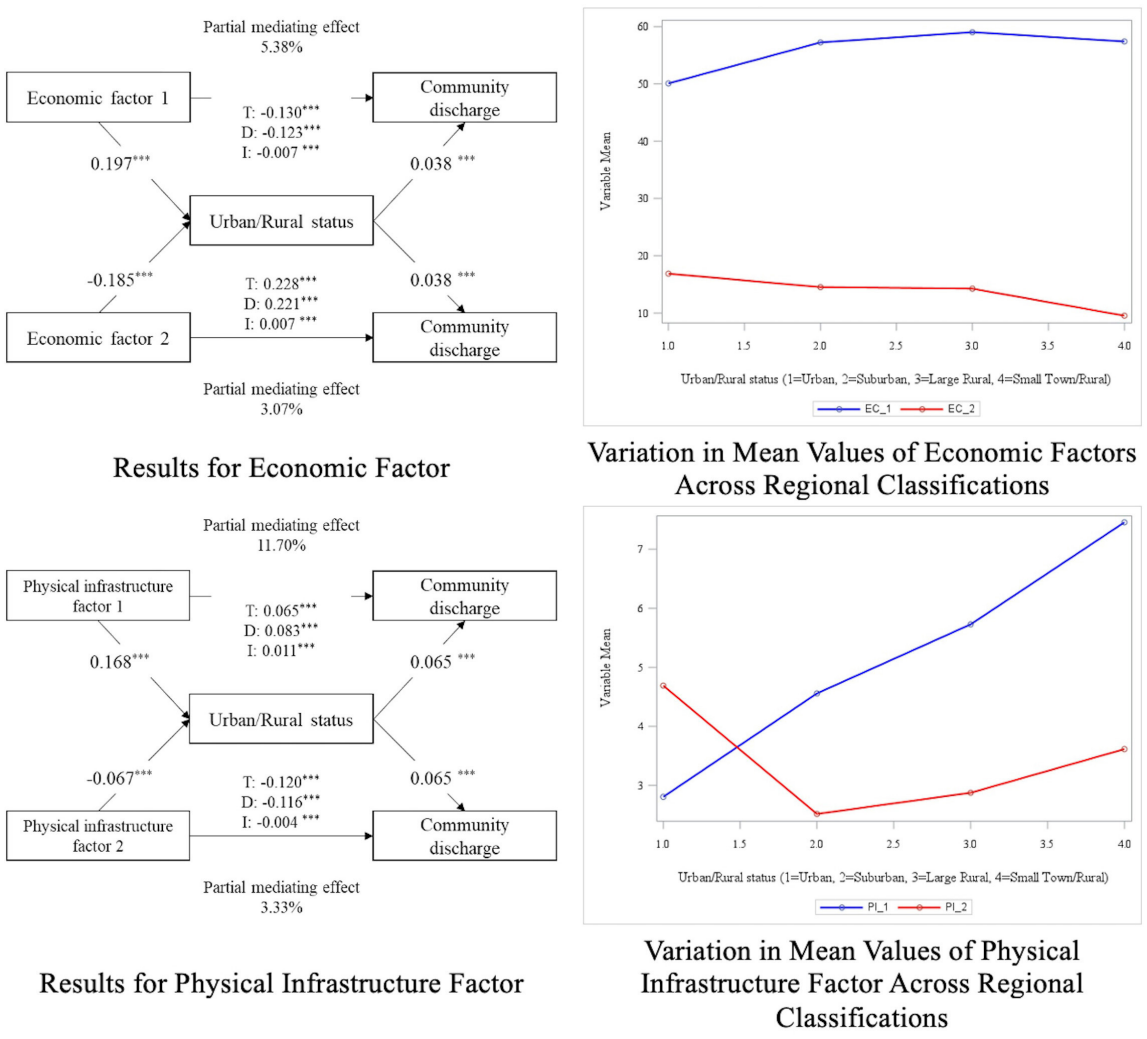
Diagram of mediation analysis results (factors with opposite outcomes). *p* > 0.05*, *p* > 0.001**, *p* > 0.0001***.

**Figure 2:**
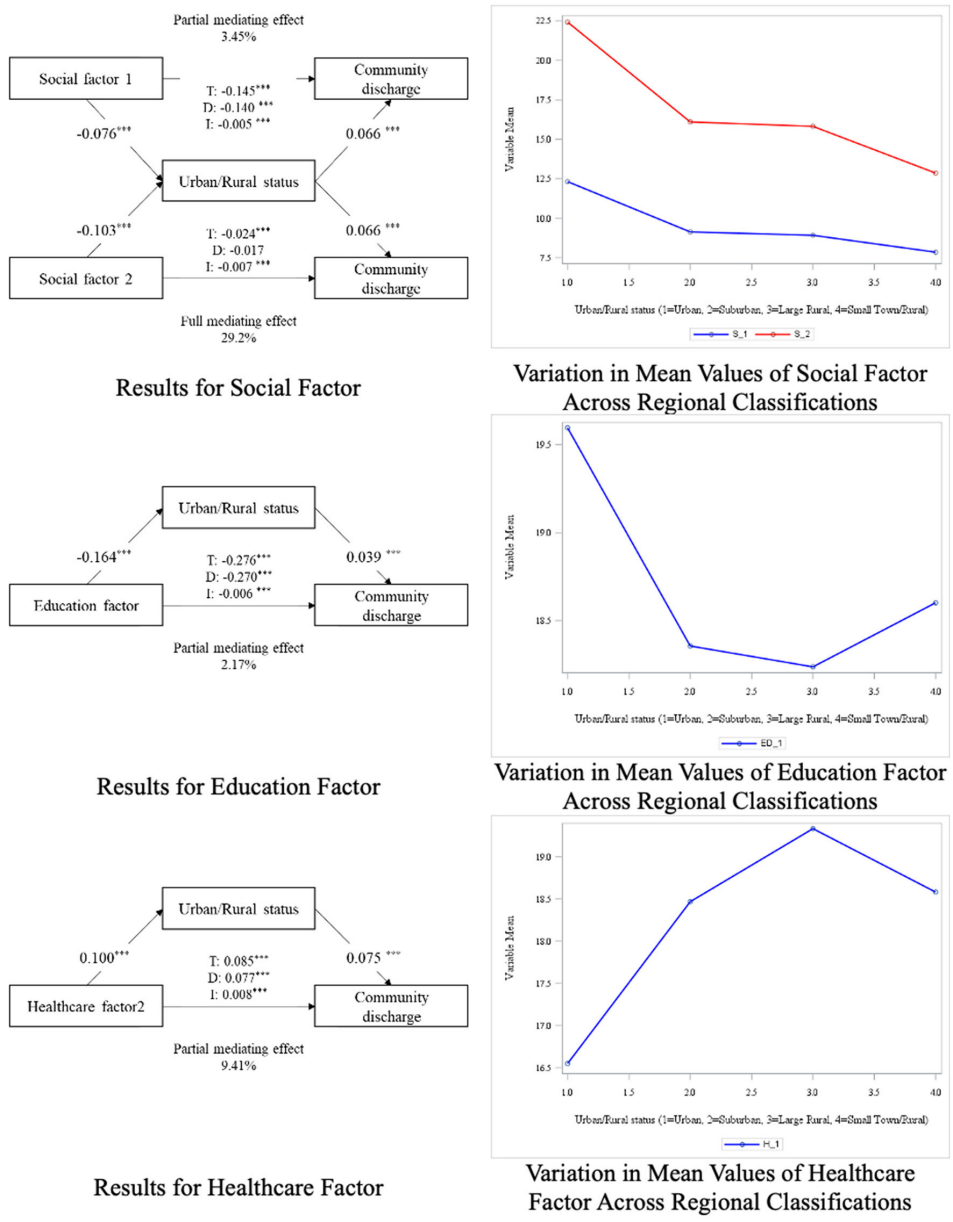
Diagram of mediation analysis results (factors with similar outcomes). *p* > 0.05*, *p* > 0.001**, *p* > 0.0001***.

Table 3 presents the results of the SEM mediation analysis, which investigated the mediating effect of RUCA on the relationship between the nine factors and CD rate. The diagrams in Figures 1, 2 provide a visual representation of these effects, with the left panels depicting the structural pathways for each factor's mediating impact through RUCA. Complementing these, the right-side graphs illustrate distinct patterns in the relationship outcomes: Figure 1 highlights contrasting values across factors, showcasing opposing trends, while Figure 2 underscores the aligned values, indicating consistent directional relationships among the factors. The study found that, with the exception of the second social factor (*p* = 0.174), all direct effects between the factors and CD rate showed significant differences. Additionally, significant differences were observed in the relationships between all factors and region, as well as between all regions and CD rate. This indicates that region acts as a partial mediator for all factors except the second social factor. For the first social factor, the indirect effect was −0.005, with a total effect of −0.145, indicating that region accounted for 3.45% of the partial mediation effect (*RMSE* = 0.059, *CFI*/*TLI* = 0.929/0.875, and *SRMR* = 0.029). For the first economic factor, the indirect effect was −0.007, with a total effect of −0.123, showing that region accounted for 5.38% of the partial mediation effect. The second economic factor showed an indirect effect of 0.007 and a total effect of 0.221, with region mediating 3.07% of the effect (*RMSE* = 0.024, *CFI*/*TLI* = 0.941/0.876, and *SRMR* = 0.015). For the education factor, the indirect effect was −0.006, with a total effect of −0.276, indicating that region accounted for 2.17% of the partial mediation effect (*RMSE* = 0.030, *CFI*/*TLI* = 0.982/0.956, and *SRMR* = 0.008). For the first physical infrastructure factor, the indirect effect was 0.011, with a total effect of 0.094, showing that region had the highest partial mediation effect at 11.70%. The second physical infrastructure factor had an indirect effect of −0.067 and a total effect of −0.116, with region accounting for 3.33% of the partial mediation effect (*RMSE* = 0.041, *CFI*/*TLI* = 0.916/0.869, and *SRMR* = 0.051). In the case of the two healthcare factors, the initial analysis aimed to examine both factors together. However, due to a negative residual variance estimate in the H3 item of the first factor, which caused model fit issues, the first factor was excluded. The final analysis proceeded with only the second healthcare factor, which showed an indirect effect of 0.008 and a total effect of 0.085, with region mediating 9.41% of the effect (*RMSE* = 0.024, *CFI*/*TLI* = 0.973/0.840, *SRMR* = 0.006).

**Table 3:** Mediation analysis results of residence area between social determinants of health factor and community discharge.

**Pathway**	**Standardized coefficient (β)**	**SE**	***p*-value**
S_1 → CD	−0.140	0.005	< 0.0001
S_2 → CD	−0.007	0.005	0.174
S_1 → Region	−0.076	0.013	< 0.0001
S_2 → Region	−0.103	0.012	< 0.0001
Region → CD	0.066	0.008	< 0.0001
Model fit	RMSEA	CFI/TLI	SRMR
	0.059	0.929/0.875	0.029
EC_1 → CD	0.221	0.009	< 0.0001
EC_2 → CD	−0.123	0.009	< 0.0001
EC_1 → Region	0.197	0.026	< 0.0001
EC_2 → Region	−0.185	0.026	< 0.0001
Region → CD	0.038	0.005	< 0.0001
Model fit	RMSEA	CFI/TLI	SRMR
	0.024	0.941/0.876	0.015
ED → CD	−0.270	0.005	< 0.0001
ED → Region	−0.164	0.018	< 0.0001
Region → CD	0.039	0.005	< 0.0001
Model fit	RMSEA	CFI/TLI	SRMR
	0.030	0.982/0.956	0.008
PI_1 → CD	0.083	0.007	< 0.0001
PI_2 → CD	−0.116	0.006	< 0.0001
PI_1 → Region	0.168	0.019	< 0.0001
PI_2 → Region	−0.067	0.010	< 0.0001
Region → CD	0.065	0.008	< 0.0001
Model fit	RMSEA	CFI/TLI	SRMR
	0.041	0.916/0.869	0.051
H_2 → CD	0.077	0.009	< 0.0001
H_2 → Region	0.100	0.011	< 0.0001
Region → CD	0.075	0.009	< 0.0001
Model fit	RMSEA	CFI/TLI	SRMR
	0.024	0.973/0.840	0.006

CD, community discharge, EC, economic, ED, education, H, healthcare, PI, physical infrastructure, S, social.

## Discussion

The primary aim of this study was to explore the relationship between community-level SDH and CD rates. Through EFA and CFA, we identified five major SDH contexts: social, economic, education, physical infrastructure, and healthcare similar to those used by the WHO and healthy people 2030 (3, 7). These factors were analyzed for their impact on CD rates across different urban/rural categories: urban core area, suburban area, large rural area, and small town/rural area. Our results indicate significant variability in CD rates based on community-level SDH, with factors such as economic deprivation, social inequality, and lack of healthcare access emerging as critical barriers to CD.

The findings from the SEM regression analysis underscore the significant impact of community social structures on CD outcomes. Higher percentages of single-parent families, minority populations, and foreign-born residents were linked to lower CD rates. This aligns with existing research that highlights how social fragmentation and minority status contribute to poorer health outcomes, often through increased stress, reduced access to healthcare, and limited social support (50). Furthermore, the mediation analysis revealed that urban/rural categories partially mediated the relationship between social factors and CD rates. Urban core areas with higher percentages of foreign-born residents and non-citizens showed lower CD rates. This is consistent with prior research demonstrating that geographic disparities in healthcare access disproportionately impact minority communities, exacerbating health disparities (51). Additionally, the complex relationship between language proficiency and health outcomes was evident in the data. Urban areas with a higher proportion of individuals with limited English proficiency exhibited lower CD rates. This supports previous studies showing that language barriers can obstruct effective communication between patients and healthcare providers, ultimately affecting the quality of care and discharge planning (52, 53).

Economic factors play a complex role in influencing CD rates, particularly in the context of poverty and labor force non-participation. According to our SEM regression analysis, increased levels of poverty and labor force non-participation are associated with a significant decrease in CD rates. This finding underscores the connection between economic hardships and limited access to essential healthcare services in general (54, 55), particularly following rehabilitation. This finding also aligns with existing research at the individual level highlighting that populations experiencing economic instability often face barriers to healthcare which leads to poor outcomes (56). In contrast, the mediation analysis indicates distinct trends across urban and rural areas. Specifically, higher poverty rates among minority populations correlate with a decrease in CD rates and are more prevalent in urban settings, suggesting that urban areas face unique challenges related to economic hardship, where healthcare infrastructure and resources are often strained (57, 58). This urban context likely exacerbates the negative impact of economic disadvantages on rehabilitation outcomes, as these communities may struggle with an overcrowded healthcare systems and/or limited access to essential services. Conversely, increased poverty and labor force non-participation in rural areas appear to be associated with an increase in CD rates, suggesting that rural areas may benefit from stronger community networks or targeted support initiatives that help counterbalance economic challenges (59, 60).

The education context also had a significant impact on CD rates. This study found that higher proportions of individuals with advanced education were associated with lower CD rates. Individuals with higher education levels generally have greater access to social and economic resources, and this finding suggests that they may be more likely to utilize healthcare following rehabilitation potentially due to their access to greater resources. Notably, the negative association between higher education levels and CD rates was more pronounced in urban areas. Although higher educational attainment is generally associated with improved health outcomes, prior studies have shown that individuals with greater health literacy and more complex care expectations may be more likely to seek additional services, remain within the healthcare system longer, or delay discharge planning, especially in urban contexts (61, 62). In such settings, well-educated individuals may advocate for extended rehabilitation or pursue more specialized care pathways, potentially leading to lower community discharge rates.

The physical infrastructure context was associated with CD rates. This suggests that communities where residents are reliant on public transportation, regardless of urban/rural status, may see significant challenges in achieving successful reintegration into the community. These findings are consistent with previous research demonstrating the negative impact of limited transportation accessibility on health outcomes (63). For those dependent on public transit, accessing healthcare services post-discharge can be particularly constrained, posing a major barrier to recovery during the rehabilitation process (64). Additionally, the lack of transportation accessibility can diminish patients' ability to live independently, further hindering their reintegration into the community. In the mediation analysis, areas with a higher proportion of individuals who use public transportation or walk to work, typically urban regions, were associated with lower CD rates. This may reflect the challenges of navigating healthcare in urban environments, where access to transportation services plays a crucial role in post-discharge recovery (63). These findings underscore the importance of improving transportation access to promote better health outcomes, particularly in underserved communities (65). Additionally, we found that areas with housing lacking complete kitchen and plumbing in rural areas were associated with an increase in CD rates in the mediation analysis, meaning higher successfully discharged to the community in areas with limited facilities. This is a counterintuitive finding as one might expect that housing unit challenges in rural areas would hinder CD. However, this finding may be explained by the strength of informal caregiving networks and supports that are more prevalent in rural areas. In these rural areas, despite inconsistent housing characteristics, family members or neighbors may provide essential support that facilitates successful reintegration into the community (66). These findings suggest that rural areas may have unique social and healthcare dynamics that enable successful CD even under challenging circumstances.

The healthcare context also played a crucial role in determining CD rates. In this study, a higher proportion of individuals covered by TRICARE or VA health insurance was associated with increased CD rates. This suggests that individuals with military or veteran' insurance may receive more comprehensive and coordinated care that supports successful reintegration into the community. This finding aligns with existing research showing that access to comprehensive healthcare coverage improves health outcomes, particularly in vulnerable populations (67). The mediation analysis further revealed that areas with higher proportions of individuals covered by TRICARE or VA insurance are more likely to be in rural areas, where CD rates tend to be higher. This highlights the importance of healthcare coverage in enhancing discharge outcomes, particularly in rural settings where TRICARE or VA insurance is more prevalent (68). Rural areas may benefit from stronger community networks and tailored healthcare resources, which can positively influence CD rates. These results underscore the necessity of expanding access to comprehensive healthcare coverage, especially in rural regions, to improve rehabilitation outcomes across diverse populations (69).

Although this study is based on U.S.-specific geographic areas, such as rehabilitation service areas (RSAs) and ZIP code tabulation areas (ZCTAs), the underlying methodological approach is transferable. Our framework links area-level social determinants of health to rehabilitation outcomes through geographic mediators. This approach can be applied in other health systems that utilize small-area geographies and population-based data. Countries like the United Kingdom, Canada, and Australia maintain similar administrative structures in healthcare planning and service delivery. In these settings, comparable methods could be used to explore equity in post-acute care access and outcomes.

### Limitations

There are certain limitations in this study that warrant attention. First, the merging of the RSA crosswalk dataset with its 1,711 records with over 40,000 AHRQ records, linked using the ZCTA contextual variables, resulted in a nested data structure. However, because the CD rate variable was calculated based on RSAs, we were unable to apply multilevel modeling for CD as the outcome variable. To address this, we applied SEM regression and mediation analysis to ensure analytical rigor despite the limitation. Additionally, our model incorporated five SDH domains and nine factors derived from EFA and CFA, making the analysis inherently complex. This complexity, combined with the inclusion of CD rate as the outcome and RUCA categories as a mediator, led to some model fit indices falling below standard thresholds. However, we mitigated this by refining factor loadings and iterating the model to optimize its fit. Given the theoretical importance of preserving distinct SDH domains, we retained the full nine-factor structure despite moderate CFI and TLI values. Previous research has shown that complex models with large sample sizes and multiple latent constructs often yield lower comparative fit indices, yet still provide valid insights when supported by conceptual coherence and acceptable RMSEA and SRMR values. Since this analysis was conducted on the contextual/ecological level, it is important to not interpret these findings on the individual level. Additionally, although exploration and confirmatory factor analyses were conducted at the ZCTA level (*n* = 40,476) to leverage the variability and maintain statistical power, the outcome variable, community discharge rate, was aggregated at the RSA level (*n* = 1,711). This analytical misalignment between the level of measurement for independent and dependent variables may limit the direct interpretability of latent factors at the outcome level.

## Conclusion and future directions

Our analysis demonstrated that area-level social determinants of health relate to community discharge outcomes following post-acute rehabilitation. Moreover, these relationships have differing impacts in rural and urban areas. Urban/rural areas mediated the relationship between social, economic, educational, physical infrastructure and healthcare factors and successful community discharge rates. This highlights how geographic context shapes the impact of these factors on rehabilitation outcomes. These finding confirm the multifaceted connection between area-level social determinants of health and community discharge following rehabilitation. Therefore, careful consideration of drivers of community integration and healthcare use in rural and urban areas is warranted. Future studies of rural and urban outcome differences across RSAs could benefit from exploring more advanced analytical techniques, such as multilevel SEM, to improve model precision and better inform public health approaches. Despite these challenges, our study provides valuable insights into how area-level contexts (social, economic, educational, physical infrastructure, and healthcare) influence community discharge outcomes following post-acute care rehabilitation.

## Data Availability

Publicly available datasets were analyzed in this study. This data can be found here: Agency for Healthcare Research and Quality available at: https://www.ahrq.gov/sdoh/data-analytics/sdoh-data.html; Rehabilitation Service Areas (for post-acute care), doi: https://doi.org/10.5281/zenodo.14291764.
