# Supplementary material for: Social determinants of health and rehabilitation service areas: an urban and rural mediation analysis
Source: Front Public Health. 2025 Jun 18;13:1562610. doi: 10.3389/fpubh.2025.1562610 (PMC12213588; doi:10.3389/fpubh.2025.1562610)
Supplement: Supplementary file 1 [file Table_1.docx]

Appendix 1. Descriptive for 34 selected ACS variables representing ZCTA-level percentages

| Factor | Variable | Description |
| --- | --- | --- |
| Social | S1 | % of population with a disability |
|  | S2 | % of population that is male |
|  | S3 | % of population ages 65 and over |
|  | S4 | % of families with children that are single-parent families |
|  | S5 | % of population that does not speak English well or at all |
|  | S6 | % of population that is minority |
|  | S7 | % of householders who minority |
|  | S8 | % of children living with grandparent householder whose grandparent is responsible for them |
|  | S9 | % of population that is foreign-born |
|  | S10 | % of population who are not U.S. citizens |
| Economic | EC1 | % of population under 3.99 of the poverty threshold |
|  | EC2 | % of population with household income less than $24,999 |
|  | EC3 | % of population not in labor force |
|  | EC4 | % of Asian population below poverty level |
|  | EC5 | % of Black or African American population below poverty level |
|  | EC6 | % of some other race population below poverty level |
| Education | ED1 | % of population with a bachelor’s degree |
|  | ED2 | % of population with master’s or doctorate |
|  | ED3 | % of population with only high school diploma |
| Physical infrastructure | PI1 | % of occupied housing units without fuel |
|  | PI2 | % of population housing units lacking compete kitchen facilities |
|  | PI3 | % of population housing units lacking plumbing facilities |
|  | PI4 | % of renter-occupied housing units with rent equal to 30 percent or more of household income |
|  | PI5 | % of housing units with no vehicle available |
|  | PI6 | % of workers taking public transportation, excluding taxicab |
|  | PI7 | % of works taking taxicab, motorcycle or other means to work |
|  | PI8 | % of workers walking to work |
|  | PI9 | % of workers in households with no vehicle available |
| Healthcare | H1 | % of population other private-only health insurance combinations |
|  | H2 | % of population with direct-purchase health insurance only |
|  | H3 | % of population with Medicare, Medicaid, TRICARE/military, U.S Department of Veterans Affairs coverage |
|  | H4 | % of population with other public-only health insurance combinations |
|  | H5 | % of population with TRICARE/military or VA health insurance coverage only |
|  | H6 | % of population with no health insurance coverage |
