# Supplementary material for: Social determinants of health and rehabilitation service areas: an urban and rural mediation analysis
Source: Front Public Health. 2025 Jun 18;13:1562610. doi: 10.3389/fpubh.2025.1562610 (PMC12213588; doi:10.3389/fpubh.2025.1562610)
Supplement: Supplementary file 2 [file Table_2.docx]

Table 2. Standardized factor loadings for exploratory factor analysis models

| Constrict | Item | Factor 1 | Factor 2 | Factor 3 | | | |
| --- | --- | --- | --- | --- | --- | --- | --- |
| Social | S1 | -0.265* | 0.065* | |  |  |  |
|  | S2 | 0.049* | -0.096* | |  |  |  |
|  | S3 | -0.185* | -0.193* | |  |  |  |
|  | S4 | -0.083* | 0.490* | |  |  |  |
|  | S5 | 0.860* | -0.005 | |  |  |  |
|  | S6 | 0.081* | 0.893* | |  |  |  |
|  | S7 | -0.006* | 0.969* | |  |  |  |
|  | S8 | -0.081* | 0.125* | |  |  |  |
|  | S9 | 0.913* | 0.035* | |  |  |  |
|  | S10 | 0.989* | -0.004 | |  |  |  |
| Model fit | RMSEA | CFI | TLI | | SRMR | | |
|  | .068 | .909 | .843 | | .053 | | |
| Economic | EC1 | -0.599* | 0.556* | 0.004 | | | |
|  | EC2 | 0.704* | 0.001 | 0.513* | | | |
|  | EC3 | 0.008* | 0.701* | -0.020 | | | |
|  | EC4 | -0.003 | -0.035 | 0.384* | | | |
|  | EC5 | -0.004 | 0.063* | 0.432* | | | |
|  | EC6 | -0.018 | -0.018 | 0.433* | | | |
| Model fit | RMSEA | CFI | TLI | SRMR | | | |
|  | .000 | 1.000 | 1.000 | .000 | | | |
| Education | ED2 | 0.825* |  |  | | | |
|  | ED3 | 0.784* |  |  | | | |
|  | ED4 | -0.840* |  |  | | | |
| Model fit | RMSEA | CFI | TLI | SRMR | | | |
|  | .000 | 1.000 | 1.000 | .000 | | | |
| Physical Infrastructure | PI1 | -0.033* | 0.063* |  | | | |
|  | PI2 | 0.867* | 0.020* |  | | | |
|  | PI3 | 0.947* | -0.009* |  | | | |
|  | PI4 | -0.130* | 0.124* |  | | | |
|  | PI5 | 0.011* | 0.916* |  | | | |
|  | PI6 | -0.206* | 0.664* |  | | | |
|  | PI7 | 0.136* | 0.365* |  | | | |
|  | PI8 | 0.058* | 0.492* |  | | | |
|  | PI9 | -0.006* | 0.960* |  | | | |
| Model fit | RMSEA | CFI | TLI | SRMR | | | |
|  | .039 | .950 | .905 | .026 | | | |
| Healthcare | H3 | -0.092* | 0.611* |  | | | |
|  | H4 | -0.397* | 0.044* |  | | | |
|  | H5 | 0.902* | 0.005* |  | | | |
|  | H6 | 0.232* | -0.077* |  | | | |
|  | H7 | 0.006* | 0.969* |  | | | |
|  | H8 | 0.339* | -0.165* |  | | | |
| Model fit | RMSEA | CFI | TLI | SRMR | | | |
|  | .035 | .937 | .763 | .017 | | | |

*: Statistical significance at the 0.05 level.
