# Supplementary material for: Social determinants of health and rehabilitation service areas: an urban and rural mediation analysis
Source: Front Public Health. 2025 Jun 18;13:1562610. doi: 10.3389/fpubh.2025.1562610 (PMC12213588; doi:10.3389/fpubh.2025.1562610)
Supplement: Supplementary file 3 [file Table_3.docx]

Appendix 3. Confirmatory factor analysis results

| Variable | Social_1 | Social_2 | | Economic_1 | Economic_2 | Education | | Physical infrastructure_1 | | Physical infrastructure_2 | | Healthcare_1 | Healthcare_2 | |  |
| --- | --- | --- | --- | --- | --- | --- | --- | --- | --- | --- | --- | --- | --- | --- | --- |
| S5 | 0.859 |  | |  |  |  | |  | |  | |  |  | |  |
| S9 | 0.929 |  | |  |  |  | |  | |  | |  |  | |  |
| S10 | 0.985 |  | |  |  |  | |  | |  | |  |  | |  |
| S4 |  | 0.459 | |  |  |  | |  | |  | |  |  | |  |
| S6 |  | 0.912 | |  |  |  | |  | |  | |  |  | |  |
| S7 |  | 0.977 | |  |  |  | |  | |  | |  |  | |  |
| EC1 |  |  | | 0.834 |  |  | |  | |  | |  |  | |  |
| EC3 |  |  | | 0.445 |  |  | |  | |  | |  |  | |  |
| EC4 |  |  | |  | 0.380 |  | |  | |  | |  |  | |  |
| EC5 |  |  | |  | 0.442 |  | |  | |  | |  |  | |  |
| EC6 |  |  | |  | 0.434 |  | |  | |  | |  |  | |  |
| ED1 |  |  | |  |  | 0.850 | |  | |  | |  |  | |  |
| ED2 |  |  | |  |  | 0.801 | |  | |  | |  |  | |  |
| ED3 |  |  | |  |  | -0.794 | |  | |  | |  |  | |  |
| PI2 |  |  | |  |  |  | | 0.884 | |  | |  |  | |  |
| PI3 |  |  | |  |  |  | | 0.931 | |  | |  |  | |  |
| PI5 |  |  | |  |  |  | |  | | 0.945 | |  |  | |  |
| PI6 |  |  | |  |  |  | |  | | 0.637 | |  |  | |  |
| PI7 |  |  | |  |  |  | |  | | 0.378 | |  |  | |  |
| PI8 |  |  | |  |  |  | |  | | 0.491 | |  |  | |  |
| PI9 |  |  | |  |  |  | |  | | 0.930 | |  |  | |  |
| H1 |  |  | |  |  |  | |  | |  | | 0.572 |  | |  |
| H5 |  |  | |  |  |  | |  | |  | | 0.989 |  | |  |
| H3 |  |  | |  |  |  | |  | |  | |  | 0.599 | |  |
| H6 |  |  | |  |  |  | |  | |  | |  | 0.428 | |  |
| Model fit |  | | RMSEA | | | | CFI | | TLI | | SRMR | | |  |  |
|  |  | | .058 | | | | .803 | | .754 | | .075 | | |  |  |
